# Trends in the management levels of metabolic risk factors in middle-aged and elderly patients with type 2 diabetes mellitus: The Korean National Health and Nutrition Examination Survey 1998–2014

**DOI:** 10.1371/journal.pone.0189361

**Published:** 2017-12-11

**Authors:** Sukyung Cho, Haeun Jang, Kyong Park

**Affiliations:** Department of Food & Nutrition, Yeungnam University, Gyeongbuk, Korea; Medizinische Universitat Innsbruck, AUSTRIA

## Abstract

The present study examined the temporal trends in the management of blood pressure, glucose, and lipid levels among middle-aged and elderly patients with type 2 diabetes using data from the Korean National Health and Nutrition Examination Survey (1998–2014). A total of 3,689 patients with diabetes were included and divided into middle-aged (30–64 years old) and elderly (≥65 years old) groups. Demographic and lifestyle data were obtained using a self-reported questionnaire, and trained medical staff obtained fasting blood samples and blood pressure data. Laboratory testing was performed to determine blood glucose, cholesterol, and triglyceride levels. In the multivariable adjusted models, significant decreasing trends in the prevalences of hyperglycemia and hypertension were observed in both age groups during 1998–2014, although no trends were observed for lipid levels. Based on the most recent survey, approximately 50% of patients with diabetes had hyperglycemia, and one-third of patients with diabetes and hypertension did not appropriately manage their blood pressure. In addition, 50% of the patients in both age groups did not manage their lipid profiles, and the management of lipid profiles did not improve in recent years. These results suggest that continuous follow-up is needed to effectively manage metabolic risk factors, especially lipid profiles, among patients with diabetes.

## Introduction

Type 2 diabetes mellitus (T2DM) is a major chronic disease that is strongly associated with aging, with increasing prevalence and incidence at older ages [1, 2]. A national statistical report regarding diabetes from the Centers for Disease Control and Prevention states that diabetes affects >1 billion adults who are ˃65 years old, which corresponds to approximately 25% of this population [3]. In 2050, the number of >65-year-old patients with diabetes is expected to exceed 2.6 billion [4]. According to the Korean National Health and Nutrition Examination Survey (KNHANES), the prevalence of diabetes is 23.0% among Koreans who are ˃65 years old, which is similar to the rates from other countries [5, 6]. In addition, approximately 25.8% of the elderly population has glucose intolerance, and 50% of elderly adults have been diagnosed with diabetes or are likely to develop diabetes, according to the most recently published KNHANES findings [5].

The diagnosis and treatment of T2DM among elderly people is similar to that among younger people [7–9]. However, elderly patients have elevated risks of diabetes complications, including cardiovascular disease, retinopathy, nephropathy, and amputation of the toes, feet, or legs [10, 11]. Moreover, physical disabilities and other health conditions should be considered in the management of diabetes, as older age is accompanied by declines in physical and cognitive activity, mental and behavioral health (including depression, Alzheimer’s disease, social isolation, and living alone), diet, vision, and ability to urinate [6, 7, 9]. Among elderly adults, it is especially important to maintain constant values for blood glucose concentration, blood pressure, and lipid profile, which can help prevent diabetes complications and decrease the mortality rate [12, 13]. Therefore, comprehensive management with appropriate control of blood glucose concentration, blood pressure, and lipid levels are needed to address T2DM in the elderly population [6, 9].

Previous studies have demonstrated that older patients with T2DM are more likely to achieve fasting plasma glucose control and glycemic control targets [14–16]. However, rigid control of glucose levels may be linked to an increased risk of hypoglycemia, which is related to impaired counterregulatory mechanisms [17]. These aging-related changes in biological mechanisms may induce complexity in the management of T2DM in the elderly population. Furthermore, although Western studies continue to emphasize the importance of diabetes management in the elderly population, few studies have examined Asian populations. Thus, the present study assessed the overall lifestyle and dietary management among elderly Korean patients with T2DM, and examined their blood glucose, blood pressure, and lipid management patterns based on KNHANES I–VI data (1998–2014).

## Materials and methods

The KNHANES is an ongoing national cross-sectional survey in South Korea that uses a complex, stratified, multistage, probability-cluster sampling [18]. The initial KNHANES were conducted every 3–4 years (I: 1998, II: 2001, and III: 2005), although KNHANES IV (2007–2009) began using an annual survey with a rolling sample system that is generated every 3 years to avoid seasonal bias. Probability sampling was performed using 20 households from 192 regions, and approximately 10,000 individuals who were ≥1 year old were included.

### 1. Population

**Fig 1:**
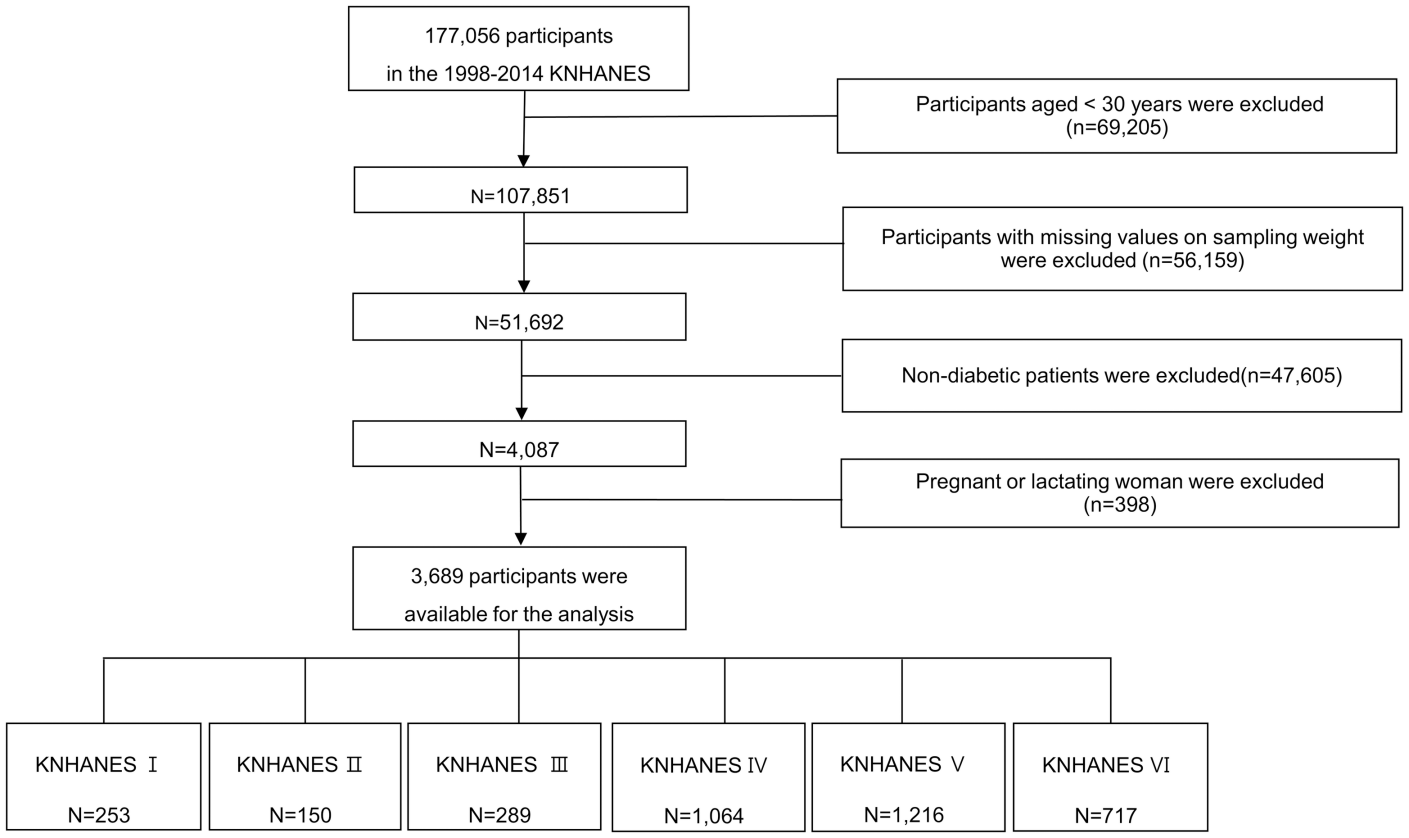
Study flow chart using data from the Korean National Health and Nutrition Examination Survey (1998–2014). The final participants (3,689 patients) was selected from 177,056 individuals who participated in the six surveys, and the participants for each KNHANES survey are shown.

The present study examined data sets from KNHANES I (1998) up to the second period of KNHANES VI (2014). Only patients with confirmed T2DM were considered eligible, and these cases were identified based on a self-reported medical history of diabetes (based on a physician’s diagnosis) or medical treatment using insulin or oral hypoglycemic medication. Among the 4,087 patients with T2DM who were ≥30 years old in the 1998–2014 surveys (excluding cases with missing sampling weight data), we excluded 398 women who were pregnant or breastfeeding at the time of the survey. Thus, 3,689 patients with T2DM were included in the present study (KNHANES I: 253 patients, KNHANES II: 150 patients, KNHANES III: 289 patients, KNHANES IV: 1,064 patients, KNHANES V: 1,216 patients, and KNHANES VI: 717 patients; Fig 1).

All KNHANES participants sign an informed consent form, and the surveys were approved by the Institutional Review Board of the Korea Centers for Disease Control and Prevention (2007-02-CON-04-P, 2008-04EXP-01-C, 2009-01CON-03-2C, 2010-02CON-21-C, 2011-02CON-06-C, 2012-01EXP-01-2C, 2013-07CON-03-4C, 2013-12EXP-03-5C).

### 2. Measurements

The KNHANES involve a health examination, health interview, and nutritional survey, which provided detailed data regarding age, sex, household income, smoking status, education level, alcohol intake, psychological stress, and T2DM duration. Household income was divided into quartiles, and education level was categorized as less than high school graduation or high school graduation and above. Smoking status was classified as current smoker, previous smoker, or never smoker. Alcohol consumption was calculated by multiplying the number of alcohol servings consumed in one sitting by the frequency of consumption, and categorized as <2 or ≥2 glasses per day for men and <1 or ≥1 glass per day for women. Physical activity data (frequency and duration of vigorous activity, moderate activity, and walking) were used to calculate metabolic equivalents (METs [h/week]) [19] from the 2008–2012 surveys only. Psychosocial stress was determined based on responses to “How often do you feel stressed?”: (1) rarely (no stress), (2) sometimes (mild stress), (3) most of the time (serious stress), or (4) always (extreme stress). The duration of T2DM was categorized as <1 years, 1–2 years, 3–4 years, 5–9 years or ≥10 years. Data regarding diet therapy and use of hypolipidemic agents were collected starting with KNHANES II (2001) and KNHANES III (2005), respectively.

The health examination provided data regarding body mass index (BMI), fasting blood glucose concentration, blood pressure, total cholesterol, high-density lipoprotein cholesterol (HDL-C), low-density lipoprotein cholesterol (LDL-C), and triglyceride (TG) concentrations. All examinations were performed by professional technicians. Each participant’s BMI was calculated as kg/m^2^, and the World Health Organization’s cut-off value for Asian population was used to identify individuals who were normal/underweight (BMI of <23 kg/m^2^) or overweight/obese (BMI of ≥23 kg/m^2^) [20].

Venous blood was collected after 8 h of fasting, and the hexokinase method was used to analyze total cholesterol, HDL-C, and triglyceride levels. The analyses were performed using different equipment for the various surveys: a Hitachi 747 autoanalyzer (Hitachi, Tokyo, Japan) for KNHANES I (1998) and KNHANES II (2001), an ADVIA1650 (Siemens, Washington, DC) from 2005 to February 15, 2008, and a Hitachi Automatic Analyzer 7600 (Hitachi, Tokyo, Japan) after February 20, 2008. As LDL-C testing was not performed before 2008, Friedewald's formula [21] was used to calculate the LDL-C of participants with TG levels of >400 mg/dL. After the 2008 survey, an ADVIA1650 (Siemens, Washington, DC) system was used to measure LDL-C concentrations.

Blood pressure was measured after a period of relaxation in the seated position using a mercury sphygmomanometer. Baumanometer^®^ Desk model 0320 (W.A. Baum, New York, NY) was used for KNHANES I–V and a Baumanometer^®^ Wall Unit 33 (0850) (W.A. Baum, New York, NY) was used after KNHANES V. Blood pressure data from the second and third periods of KNHANES IV were adjusted for differences in arm heights that were used during the measurements.

### 3. Definitions of variables

The major variables of interest were hyperglycemia (a fasting blood glucose level of ≥126 mg/dL) [8] and hypertension (a blood pressure of >140/90 mmHg, using antihypertensive drugs, or a physician diagnosis of hypertension) [22]. The management of hypertension was evaluated based on “uncontrolled blood pressure”, which was defined as a blood pressure of >140/90 mmHg and the absence of anti-hypertensive drug therapy or diet/lifestyle therapy (e.g., adherence to recommended dietary and lifestyle modifications to control blood pressure). Dyslipidemia was identified based on a total cholesterol (TC) level of ≥240 mg/dL, an HDL-C level of <40 mg/dL, an LDL-C level of ≥160 mg/dL, a TG level of ≥ 200 mg/dL, the use of lipid-lowering drugs, or a physician diagnosis of dyslipidemia [23]. The management of lipid disorders was evaluated based on “uncontrolled lipid profiles”, which were defined as having high levels of lipids (TC of ≥240 mg/dL, HDL-C of <40 mg/dL, LDL-C of ≥160 mg/dL, or TG of ≥200 mg/dL) and the absence of lipid-lowering drug therapy or diet/lifestyle therapy.

### 4. Statistical analysis

All statistical analyses were performed considering the KNHANES’ complex survey design, sampling weights, and stratified and clustered sampling approach. Participants were categorized into middle-aged (30–64 years old) and elderly (≥65 years old) groups. The weighted frequencies of categorical variables and weighted means and standard errors of continuous variables were calculated using SAS SURVEY procedures. Potential confounding factors were considered based on previous research and the results of the descriptive analysis. In addition, effect modification in the multivariable model was tested for these factors using multiplicative terms. Multivariable generalized linear regression analysis was used to estimate adjusted mean and 95% confidence interval (CI) values for the prevalences of hyperglycemia, uncontrolled high blood pressure, hypertension, uncontrolled lipid profiles, and dyslipidemia in both age groups and for each survey. The *P*-values for trends were calculated using the median value of the prevalence of metabolic disorder as a continuous variable in each survey during the 16-year period. All analyses were performed using SAS software (version 9.4; SAS Institute, Cary, NC), and differences were considered statistically significant at a two-tailed α value of 0.05.

## Results

The general characteristics of the middle-aged and elderly groups are shown in Table 1. The elderly group had a significantly higher proportion of women, a higher household income, and a lower educational level, compared to the middle-aged group (*P* < 0.001). The proportions of overweight/obesity were high in both groups (72.7% in the middle-aged group and 67.4% in the elderly group). Compared to middle-aged individuals, elderly individuals were more likely to make healthier lifestyle choices, based on a lower proportion of never smoking (*P* < 0.001), a higher proportion of moderate alcohol consumption (*P* < 0.001), and greater adherence to the use of hypoglycemic agents (*P* < 0.001). However, the elderly group had a lower rate of physical activity than the middle-aged group (*P* < 0.001). The duration of diabetes was associated with age (*P* < 0.001), and the elderly group tended to have a longer duration of diabetes, compared to the middle-aged group.

**Table 1:** Characteristics of patients with diabetes mellitus according to age group.

	<65 years	≥65 years	*P*-value ^1^
**N**	1,861	1,828	
**Median age (years)**	57 (30–64)	71 (65–93)	
**Male**	994 (53.4)	777 (42.5)	<0.001
**Income** ^2^			
**Low**	542 (29.5)	420 (23.4)	<0.001
**Mid-low**	497 (27.0)	459 (25.5)	
**Mid-high**	390 (21.2)	463 (25.8)	
**High**	411 (22.3)	455 (25.3)	
**Smoking status** ^2^			
**Current smoker**	485 (26.1)	251 (13.8)	<0.001
**Previous smoker**	427 (23.0)	518 (28.5)	
**Never smoker**	945 (50.9)	1,047 (57.7)	
**High school or higher education**	848 (45.7)	381 (20.9)	<0.001
**Overweight or obesity** ^3^	1,349 (72.7)	1,226 (67.4)	<0.001
**Moderate alcohol consumption** ^4^	1,112 (60.0)	1,426 (78.5)	<0.001
**METs** ^2^			
**<20**	561 (47.3)	738 (57.5)	<0.001
**20–39**	253 (21.3)	252 (19.6)	
**≥40**	373 (31.4)	294 (22.9)	
**Stress status** ^2^			
**Extreme stress**	113 (6.1)	99 (5.5)	<0.001
**Serious stress**	439 (23.6)	331 (18.2)	
**Mild stress**	978 (52.7)	780 (43.0)	
**No stress**	327 (17.6)	606 (33.4)	
**Diabetes treatment**			
**Oral hypoglycemic agents**	1,440 (77.4)	1,552 (84.9)	<0.001
**Diet therapy**	751 (40.4)	791 (43.3)	0.07
**Diabetes duration (years)**^2^			
**<1**	158 (9.2)	100 (6.0)	<0.001
**1–2**	409 (23.7)	249 (14.9)	
**3–4**	304 (17.6)	268 (16.0)	
**5–9**	443 (25.7)	434 (25.9)	
**≥10**	411 (23.8)	623 (37.2)	

Data are reported as number (percentage) or median (range).

^1)^ The *P*-values were calculated using the chi-square test or t-test, as appropriate.

^2)^ These categories had missing data from some cases, and the total n values are not identical.

^3)^ Body mass index of ≥23 kg/m^2^.

^4)^ Less than 1 drink/day (women) or 2 drinks/day (men).

After adjusting for sex, medication use, smoking status, moderate alcohol consumption, BMI, education level, duration of diabetes, and psychosocial stress, significant decreasing trends were detected in fasting glucose and systolic/diastolic blood pressure for both age groups during 1998–2014 (*P* for trend <0.001, Table 2). Average fasting glucose levels and diastolic blood pressure tended to be higher in the middle-aged group, while systolic blood pressure tended to be higher in the elderly group. There were no significant changes in the levels of LDL-C over time in both age groups.

**Table 2:** Fasting blood glucose, low-density lipoprotein cholesterol, and systolic/diastolic blood pressure among patients with diabetes mellitus according to age group and survey period.

KNHANES	Systolic blood pressure (mmHg)		*P*-value	Diastolic blood pressure (mmHg)		*P*-value
	<65 years	≥65 years		<65 years	≥65 years	
I	144.2 ± 2.8	146.3 ± 3.6	0.5	84.0 ± 1.5	77.4 ± 1.7	<0.0001
II	137.2 ± 4.1	136.9 ± 3.3	0.9	82.9 ± 2.2	78.2 ± 1.9	0.02
III	127.4 ± 2.1	135.6 ± 2.7	0.001	79.7 ± 1.1	75.6 ± 1.4	0.01
IV	122.6 ± 1.1	126.9 ± 1.2	<0.0001	77.4 ± 0.7	73.6 ± 0.7	<0.0001
V	125.2 ± 1.3	130.6 ± 1.4	<0.0001	76.2 ± 0.7	72.5 ± 0.7	<0.0001
VI	123.8 ± 1.5	124.4 ± 1.4	0.7	76.2 ± 1.0	70.4 ± 0.8	<0.0001
*P* for trend	<0.0001	<0.0001		<0.0001	<0.0001	
KNHANES	Fasting blood glucose (mg/dL)		*P*-value	LDL-cholesterol (mg/dL)		*P*-value
	<65 years	≥65 year		<65 years	≥65 years	
I	185.9 ± 11	166.4 ± 14.5	0.07	NA	NA	
II	133.0 ± 4.4	119.3 ± 4.9	0.01	NA	NA	
III	144.2 ± 4.4	131.5 ± 5.3	0.02	110.3 ± 4.7	109.9 ± 5.7	0.9
IV	143.1 ± 3.6	130.5 ± 4.0	0.003	104.0 ± 3.9	109.2 ± 3.8	0.2
V	145.1 ± 3.8	126.6 ± 3.7	<0.0001	102.6 ± 4.5	98.3 ± 7.2	0.5
VI	140.7 ± 4.7	129.2 ± 4.4	<0.0001	102.7 ± 6.9	105.3 ± 6.2	0.7
*P* for trend	<0.0001	<0.0001		0.05	0.3	

KNHANES: Korean National Health and Nutrition Examination Survey, NA: not available. Values are adjusted for sex, medication use, smoking status (current smoker, previous smoker, or never smoker), alcohol consumption (<1 vs. ≥1 drink/day for women or <2 vs. ≥2 drinks/day for men), body mass index (<23 kg/m^2^ or ≥23 kg/m^2^), education level (less than high school or more than high school), duration of diabetes, and psychosocial stress.

**Fig 2:**
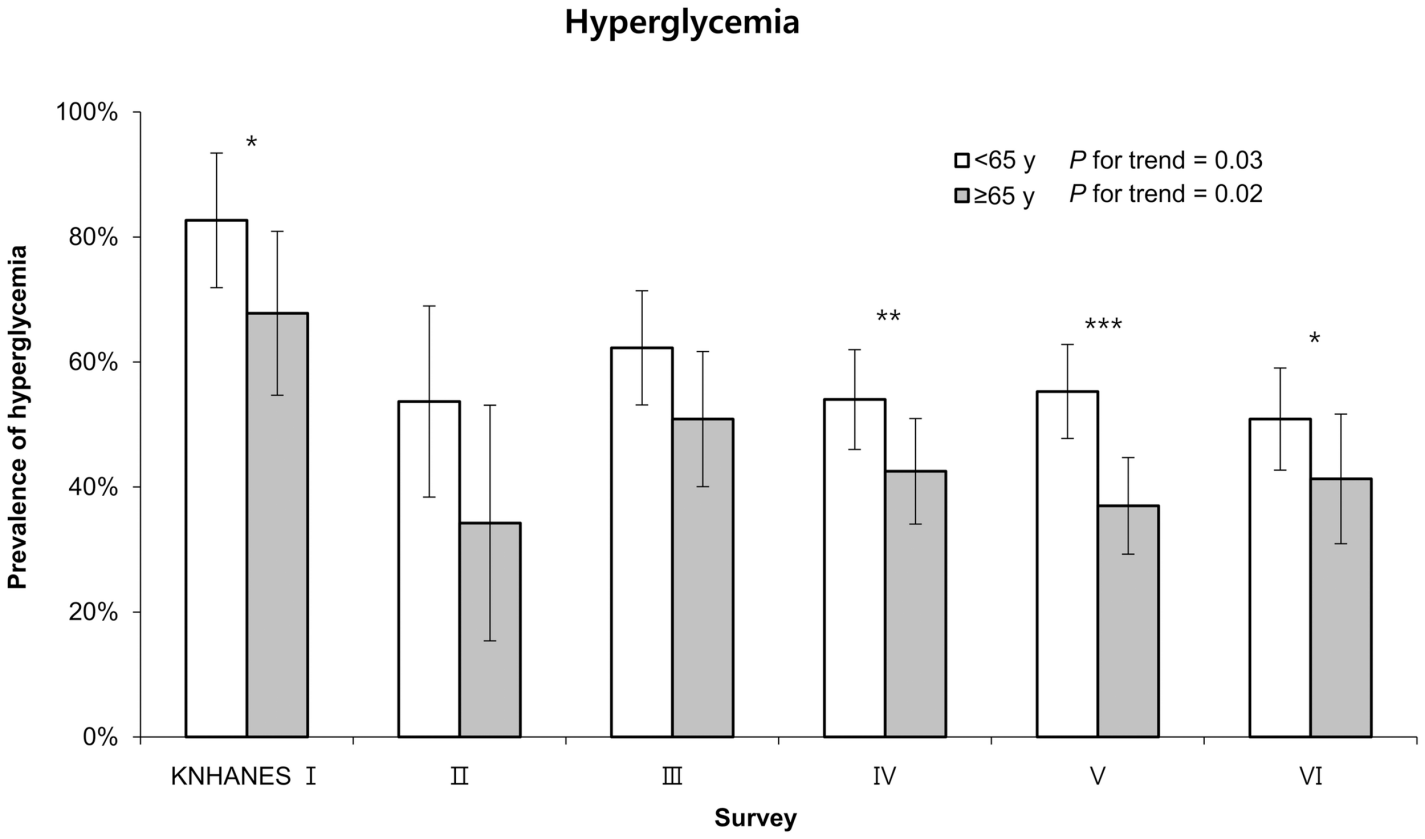
The prevalences of hyperglycemia among patients with diabetes mellitus according to age group and survey period. Values are adjusted for sex, medication use, smoking status (current smoker, previous smoker, or never smoker), alcohol consumption (<1 vs. ≥1 drink/day for women or <2 vs. ≥2 drinks/day for men), body mass index (<23 kg/m^2^ or ≥23 kg/m^2^), education level (less than high school or more than high school), duration of diabetes, and psychosocial stress. *p < 0.05, **p < 0.01, ***p < 0.001.

Fig 2 shows the prevalence of hyperglycemia among middle-aged and elderly patients with T2DM during 1998–2014, based on multivariable linear regression analysis that was adjusted for sex, medication use, smoking status, moderate alcohol consumption, BMI, education level, duration of diabetes, and psychosocial stress. The elderly group had a lower prevalence of hyperglycemia than the middle-aged group during most survey years, with the exception of KNHANES II and III, which did not reveal any significant differences between the two groups. In addition, there were significant decreasing trends in the prevalences of hyperglycemia over time in both the middle-aged and elderly groups (*P* for trend = 0.03 and 0.02 for the middle-aged and elderly groups, respectively). However, the most recent survey (KNHANES VI) revealed that approximately one-half of patients with T2DM in both age groups had hyperglycemia, which reflects unsatisfactory management of fasting blood glucose levels.

**Fig 3:**
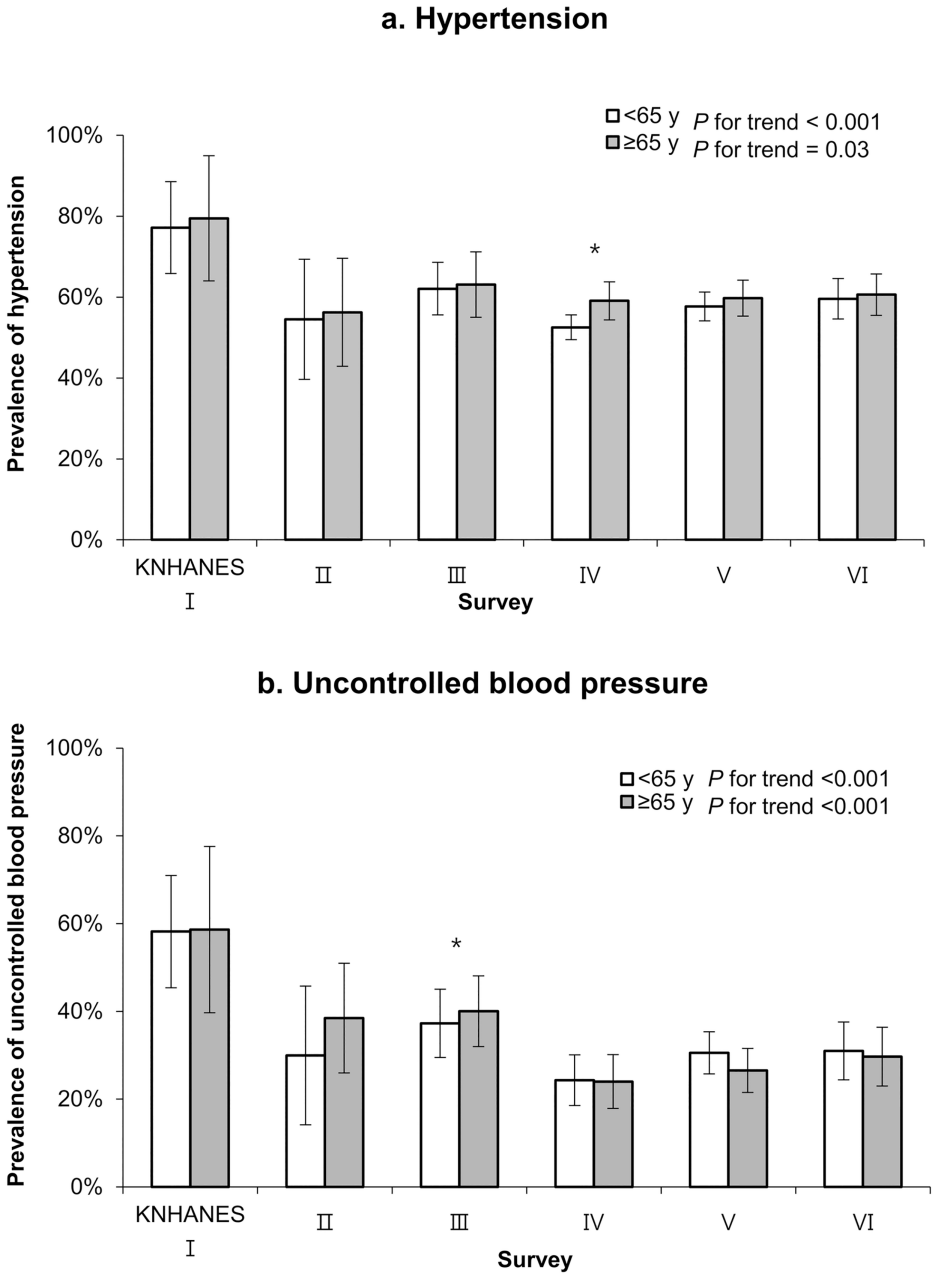
The prevalences of uncontrolled blood pressure and hypertension among patients with diabetes mellitus according to age group and survey period. Values are adjusted for sex, medication use, smoking status (current smoker, previous smoker, or never smoker), alcohol consumption (<1 vs. ≥1 drink/day for women or <2 vs. ≥2 drinks/day for men), body mass index (<23 kg/m^2^ or ≥23 kg/m^2^), education level (less than high school or more than high school), duration of diabetes, and psychosocial stress. *p < 0.05.

The only significant differences in the prevalences of uncontrolled blood pressure and hypertension between the elderly and middle-aged groups were detected during KNHANES III and IV, with significantly higher prevalences of uncontrolled blood pressure and hypertension in the elderly group. However, both groups exhibited trends towards decreasing prevalences of uncontrolled blood pressure and hypertension (uncontrolled blood pressure: middle-aged group *P* for trend <0.001, elderly group *P* for trend <0.001; hypertension: middle-aged group *P* for trend <0.001, elderly group *P* for trend = 0.03) (Fig 3). The prevalences of hypertension in the most recent survey (KNHANES VI) were 59.6% in the middle-aged group and 60.6% in the elderly group, and the prevalences of uncontrolled blood pressure were 31.0% and 29.7%, respectively.

**Fig 4:**
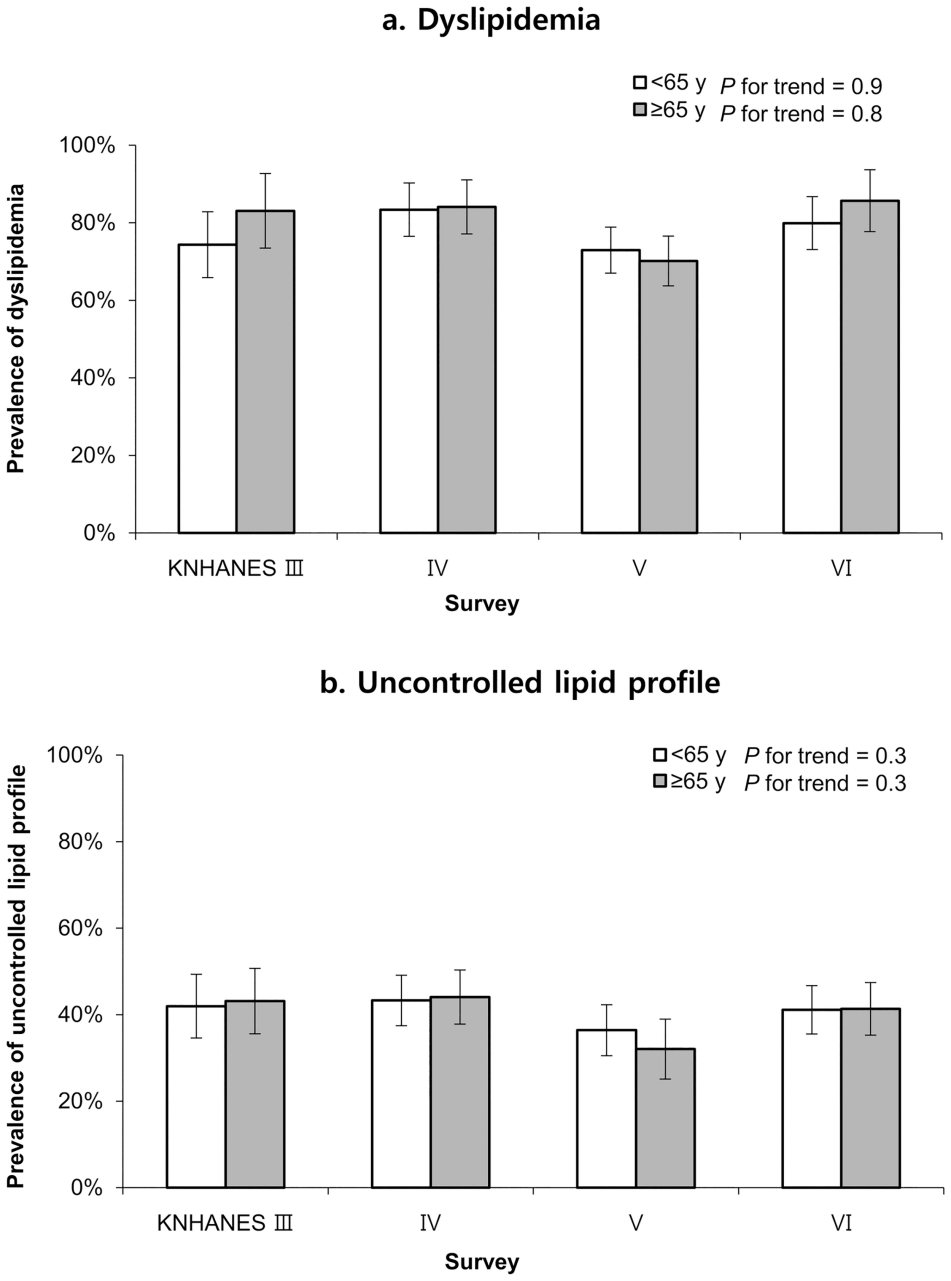
The prevalences of uncontrolled lipid profile and dyslipidemia among patients with diabetes mellitus according to age group and survey period. Values are adjusted for sex, medication use, smoking status (current smoker, previous smoker, or never smoker), alcohol consumption (<1 vs. ≥1 drink/day for women or <2 vs. ≥2 drinks/day for men), body mass index (<23 kg/m^2^ or ≥23 kg/m^2^), education level (less than high school or more than high school), duration of diabetes, and psychosocial stress.

Fig 4 shows that the prevalences of uncontrolled lipid profiles and dyslipidemia were not significantly different between the two age groups. In addition, no significant trend was observed in the prevalence of uncontrolled lipid profiles. The most recent KNHANES data revealed that the prevalences of dyslipidemia were 79.9% in the middle-aged group and 85.2% in the elderly group, and the prevalences of uncontrolled lipid profiles were 41.1% and 41.3%, respectively.

## Discussion

The present study evaluated KNHANES data from 1998–2014 for patients with confirmed T2DM who were ≥30 years old, and compared the management of blood pressure, blood glucose, and blood lipids between the middle-aged and elderly participants. Overall, one-half of the patients with T2DM in both groups did not appropriately manage their blood glucose and blood lipid levels. Furthermore, during the study period, the management of blood glucose and blood pressure appears to have improved, whereas the management of blood lipids did not improve and remains poor.

Previous studies have demonstrated that older adults have better management of blood glucose than middle-aged adults [14–16, 24–28], which is consistent with the findings of the present study. However, the management of blood glucose among elderly individuals is considered difficult because of the close relationship between aging and increased blood glucose levels. Nevertheless, older patients tend to have better diet therapy and medication adherence [29]. Although the management of blood glucose appears to be better in older adults (vs. middle-aged adults), hypoglycemic shock is a concern among older adults [30, 31]. Thus, personalized management of blood glucose levels and prevention of diabetes complications are critical for older patients with T2DM [27].

The present study revealed that blood pressure and blood lipid management levels did not differ significantly between the age groups in most survey years, although conflicting findings have been revealed in previous studies [25, 27]. For example, a cross-sectional study of 161,697 American patients with T2DM (<50 years old, 50–64 years old, 65–74 years old, and 75–85 years old) revealed that the oldest patients exhibited the poorest maintenance of normal blood pressure but the best maintenance of normal LDL-C levels [25]. Another study revealed that 20% fewer individuals who were ≥60 years old maintained blood pressures of 130/80 mmHg, compared to individuals who were <60 years old (odds ratio: 0.80, 95% CI: 0.76–0.82) [27]. Furthermore, compared to younger individuals, individuals who were ≥60 years old were 15% more likely to maintain LDL-C concentrations of <2.6 mmol/L, 21% more likely to maintain LCL concentrations of >1.1 mmol/L, and 20% more likely to maintain TG levels of <1.7 mmol/L [27]. Thus, the previous studies have suggested that elderly individuals have relatively unsatisfactory levels of blood pressure management, but satisfactory levels of blood lipid management [25, 27]. The discrepancy between the findings of the present and previous studies may be related to differences in the definitions of abnormal health conditions. For example, the present study defined hypertension as blood pressures of >140/90 mmHg, whereas the previous studies used >130/80 mmHg [27]. In addition, the definitions of dyslipidemia were based on different combinations of biomarkers. Moreover, there were differences in the statistical models and their adjustment for confounders, as well as in ethnicity and region.

The management of blood glucose levels and blood pressure among middle-aged and elderly patients with T2DM gradually improved during the study period, although the management of blood lipid profiles remained unsatisfactory. In this context, most patients with T2DM and hypertension had adequately controlled blood pressure, which was achieved using drug therapy or diet/lifestyle modification. The Korea Centers for Disease Control and Prevention have also reported that there were rapid increasing trends in the use of antidiabetic and antihypertensive drugs among patients with diabetes and hypertension, respectively. For example, the proportion of antihypertensive drug use among patients with hypertension increased from 21.9% in 1998 to 54.8% in 2007, and the proportion of insulin or oral hypoglycemic drug treatment among patients with diabetes increased from 29.7% in 1998 to 57.4% in 2007 [32]. These changes may have led to improvements in the overall management of blood glucose and blood pressure among patients with T2DM during the last 16 years, which are reflected in the findings of the present study.

However, one-half of T2DM patients with dyslipidemia had not achieved appropriate management of their blood lipid profiles. Similarly, previous studies have indicated that only one-third of T2DM patients with have adequately managed cholesterol levels, including one study of 2,591 Korean T2DM patients with dyslipidemia or hyperlipidemia [33] and another study of 4,888 patients with T2DM who were receiving statin therapy [34]. In this context, insulin resistance increases fatty acid release from adipocytes and promotes the production and release of triglycerides [35]. Many epidemiological studies have demonstrated that blood lipid profile management can reduce the risk of cardiovascular disease among patients with diabetes [36–38].

Patients with T2DM require better awareness of the importance of cholesterol and lipid management. Previous reports revealed that T2DM patients with dyslipidemia had poor diet/lifestyle therapy and medication adherence, even when their blood lipids were not properly controlled [33, 39, 40]. Moreover, cholesterol goals for patients with T2DM may be affected by patient-related factors (delays regarding medication therapy, medication adherence, and economic status), healthcare-related factors (miscommunication between the physician and patient), and medication-related factors (number of medications and adverse effects) [41]. Thus, a systemic improvement in lipid profile should be achieved through follow-up and education for patients with T2DM and dyslipidemia.

One of the strengths of the present study is the generalizability of its results, as the KNHANES provide nationally representative survey data. In addition, to the best of our knowledge, this is the first study to evaluate the management of metabolic biomarkers among patients with T2DM according to age group, and to examine the trends in these management levels during an extended period. Thus, the results of the present study may help guide the development of education and intervention strategies for patients with T2DM.

The present study also has several limitations. For example, older patients with T2DM are more likely to have a prolonged duration of disease, which is associated with management levels and personal attitudes toward diabetes. Thus, we adjusted the models for the duration of diabetes, which is a time-dependent variable, in order to minimize this bias [42, 43]. In addition, the values for blood glucose, blood pressure, and lipids were measured and analyzed using different devices and methods in the various survey years. However, the KNHANES surveys use strictly controlled and evaluated procedures to enhance the validity and reliability of the measurements and analyses [44, 45]. Another potential limitation is the possibility of residual confounding, based on the observational study design, although we attempted to minimize this issue by adjusting for confounding factors based on the findings of previous studies and our statistical analyses.

In conclusion, the present study revealed that older patients had better management of blood glucose levels, compared to middle-aged patients, although no significant differences between the age groups were observed in the management of blood pressure and lipid profiles. Furthermore, we detected trends towards improved management of blood glucose and blood pressure during the study period, although no improvement was observed for the management of blood lipid levels. Furthermore, one-half of patients with T2DM and dyslipidemia did not appropriately manage their lipid profiles, which is a serious cause for concern. These patients require continuous follow-up to effectively manage their lipid profiles. Moreover, older patients with T2DM should undergo a careful evaluation for their risk of diabetes complications and the presence of comorbidities, which can be used to help develop and implement appropriate self-management goals.
